# Traditional Chinese medicinal formula Si-Wu-Tang prevents oxidative damage by activating Nrf2-mediated detoxifying/antioxidant genes

**DOI:** 10.1186/2045-3701-4-8

**Published:** 2014-02-10

**Authors:** Mandy Liu, Ranadheer Ravula, Zhijun Wang, Zhong Zuo, Moses SS Chow, Arvind Thakkar, Sunil Prabhu, Bradley Andresen, Ying Huang

**Affiliations:** 1 Department of Pharmaceutical Sciences, College of Pharmacy, Western University of Health Sciences, Pomona, California; 2 School of Pharmacy, Faculty of Medicine, The Chinese University of Hong Kong, Shatin, New Territories, Hong Kong, China

**Keywords:** Cancer chemoprevention, Si-Wu-Tang, Z-liguistilide, ROS, Nrf2, Breast cancer, SLC7A11, HMOX1

## Abstract

**Background:**

Induction of Nrf2-mediated detoxifying/antioxidant genes has been recognized as an effective strategy for cancer chemoprevention. Si-Wu-Tang (SWT), comprising the combination of four herbs, Paeoniae, Angelicae, Chuanxiong and Rehmanniae, is one of the most popular traditional oriental medicines for women’s diseases. The purpose of this study is to determine the effects of SWT on Nrf2 pathway *in vitro* and *in vivo* and to identify the active component(s).

**Results:**

Cell viability and apoptosis were analyzed in the non-cancerous breast epithelial cell line MCF-10A after H_2_O_2_ treatment in the presence or absence of SWT using the Sulphorhodamine B assay, Annexin-V/Propidium iodide staining and flow cytometry. SWT strongly reduced H_2_O_2_ -induced cytotoxicity and apoptosis in MCF-10A cells. Expression of Nrf2 and Nrf2-regulated genes *HMOX1* (heme oxygenase 1) and *SLC7A11* (xCT) was evaluated by quantitative RT-PCR, Western Blot and immunocytochemistry. SWT strongly induced Nrf2-regulated genes at mRNA and protein levels and increased the nuclear translocation of Nrf2 in MCF-10A cells*.* The *in vivo* pharmacodynamic effect of SWT was evaluated in healthy female Sprague–Dawley rats. Short-term oral administration of SWT (1,000 mg/kg per day for six consecutive days) to rats resulted in an increased expression of Nrf2-regulated genes *Hmox1* and *Slc7A11* in the liver detected by quantitative RT-PCR. Among nine compounds that have been identified previously in the SWT products, z-liguistilide was discovered as the main component responsible for the effect of Nrf2 activation using the antioxidant response element-luciferase reporter gene assay. Z-liguistilide was confirmed with a high potency to induce Nrf2-regulated genes and Nrf2 nuclear translocation.

**Conclusions:**

Our results demonstrated that SWT and its component z-liguistilide are able to activate the Nrf2 pathway in non-cancerous cells and organs *in vitro* and *in vivo*, suggesting that SWT might be an orally effective and nontoxic agent for cancer chemoprevention.

## Background

Breast cancer is one of the most common types of cancer in women, and is the second leading cause of cancer-related deaths in the United States [1]. It is estimated that in 2013, there will be 232,340 new cases and 39,620 deaths of breast cancer [2]. Chemoprevention, using natural or synthetic agents to decrease the risk of developing invasive carcinoma, has become an important approach to reduce breast cancer morbidity and mortality [3]. Although the use of selective estrogen receptor modulators such as tamoxifen is able to prevent development of certain estrogen receptor (ER)-positive breast cancer, they have not been widely adopted as a long-term preventive strategy because of incomplete effectiveness and intolerable adverse effects [4]. In addition, they have no effect on ER-negative cancers. As breast cancer remains a global public health challenge, there is a great need for developing safe and inexpensive agents for the prevention of this disease.

The main mechanisms for breast cancer chemoprevention include hormonal modulation and limiting accumulation of genetic damage using antioxidant and anti-inflammatory agents [5]. Oxidative stress has been implicated in the processes that control carcinogenesis, aging, neurodegeneration, and many other chronic lesions [6]. In particular, the role of oxygen radicals and other reactive oxygen species (ROS) in the origin or progression of cancer has been strongly supported by extensive research [7]. Most risk factors associated with carcinogenesis of the breast, including stress, tobacco, environmental pollutants, radiation, viral infection and unhealthy diet, affect normal cells through the generation of ROS [8]. High level of ROS results in DNA damage, cell proliferation and cell invasiveness [9]. Protection of normal cells from a diversity of such events through induction of the detoxifying and antioxidant proteins is one of the promising strategies to prevent cancer and other chronic diseases [10]. This is a particularly attractive strategy for breast cancer prevention, because exposure to carcinogens, including metabolites of endogenous and exogenous estrogens, play a significant role in the development of breast cancer through the cumulative accumulation of mutagenic events over a women’s lifetime [11].

The nuclear factor erythroid 2 -related factor 2 (Nrf2), a basic zip (bZIP) transcription factor, is a key molecule that regulate many cytoprotective and antioxidant genes. Nrf2 plays a central role in the regulation of basal and/or inducible expression of the downstream genes by binding to the “antioxidant response elements” (AREs) in their promoters [12]. Nrf2 is normally sequestered in the cytoplasm by Kelch-like ECH-associated protein 1 (Keap1). When activated upon exposure to inducers, Nrf2 dissociates from Keap1, translocates to the nucleus, complexes with other nuclear factors, and binds to the ARE of many Nrf2 target genes. Supporting this notion, the Nrf2-knockout mice have been found more susceptible to carcinogenic challenges than the wild type animals [13]. Animals deficient in Nrf2 target genes such as *NQO1*, *GSTs*, or *SLC7A11* were also more susceptible to carcinogenesis [10,14-16]. Thus, there has been an increased interest to identify chemopreventive Nrf2 inducers [17]. A crucial problem is that not all the Nrf2 inducers are beneficial. Many toxic chemicals such as arsenic, quinones and chemotherapeutic drugs can also activate the Nrf2 pathways [18]. The most attractive chemopreventive agents are those that potently induce the Nrf2 dependent genes without eliciting toxic effects on normal cells [18]. Further, for long-term use as chemopreventive agent for healthy individuals, it is crucial to develop orally effective and non-toxic agents.

Si-Wu-Tang [SWT, Si-Wu decoction (Chinese name), Samultang (Korean name), or Shimotsu-to (Japanese name)], comprising the combination of four herbs, Paeoniae, Angelicae, Chuanxiong and Rehmanniae, is one of the most popular traditional oriental medicines for women’s health [19]. It has been used in Eastern Asia for about one thousand years for various women’s diseases and ranks first as the most frequently used Chinese medicines in several surveys [20]. It is an inexpensive over-the-counter preparation used for the relief of menstrual discomfort, climacteric syndrome, peri- or postmenopausal syndromes and other estrogen-related diseases [19-23]. The major principle of SWT therapy as Chinese Medicine is to improve a deficiency of Qi and Blood [24]. In various model animals, SWT has shown sedative, anti-coagulant and anti-bacterial activities, as well as protective effect on radiation-induced bone marrow damage [25,26]. Several *in vitro* and *in vivo* studies show a preventive effect of SWT on endometrial carcinogenesis induced by carcinogen and estrogen [27,28], although the mechanisms and active constituents are unknown. In addition, some decoctions of SWT series, such as Tao-Hong-Si-Wu decoction, have shown a neuroprotective activity *in vivo*[29].

The purpose of the present study is to investigate the effect of SWT on the Nrf2 pathway. Our results showed that SWT contains at least one component that is capable of activating the Nrf2 signaling pathway. Such effect was examined *in vitro* using a non-cancerous breast epithelial cell line MCF-10A and *in vivo* using healthy female Sprague–Dawley rats. This study provides a better understanding of the mechanisms of SWT as a potential antioxidant and cell protective agent and scientific evidence to support the empirical clinical use of SWT in prevention of cancer and other diseases that may involve the detrimental effects of ROS.

## Results

### Protective effects of SWT against hydrogen peroxide (H_2_O_2_) – induced cytotoxicity

**Figure 1:**
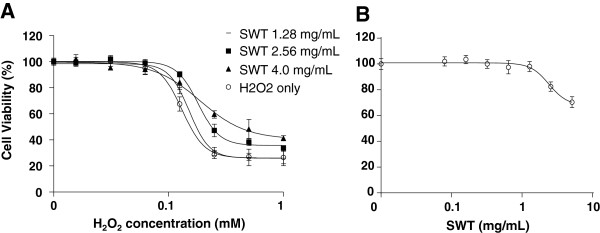
**Effects of SWT on H**_**2**_**O**_**2**_**-induced cytotoxicity in MCF-10A cells. A.** The MCF-10A cells (3,000 cells/well) were pre-treated with SWT (1.28, 2.56 or 4.0 mg/mL) for two hours then exposed to various concentrations of H_2_O_2_ for 24 hours. **B.** The cytotoxicity of SWT alone were determined on MCF-10 cells after treatment for 24 hours. Cell growth was measured using the SRB assay. Results are expressed as percentage viability of control cells without drug treatment. Points, means from three replicates; bars, S.D.

The MCF-10A cells were pre-treated with SWT extract for 2 hours before adding H_2_O_2_ and were incubated for additional 24 hours. The effect of SWT was tested in combination with various concentrations of H_2_O_2_ (0.016 to 1.0 mM). In the presence of increasing concentrations of SWT (1.28, 2.56 and 4.0 mg/mL), the IC_50_s of H_2_O_2_ were increased from 0.13 ± 0.0006 mM to 0.15 ± 0.004, 0.18 ± 0.006 and 0.19 ± 0.013 mM, respectively (Figure 1A). All the SWT and H_2_O_2_ combination treatment groups showed a statistically significant change compared to the H_2_O_2_ only group (*p* < 0.05). Treatment of MCF-10A by SWT alone at these concentrations showed moderate effect on the cell growth (the IC_50_ cannot be determined from the dose–response curve) (Figure 1B).

### Protective effects of SWT against H_2_O_2_ – induced apoptosis

**Figure 2:**
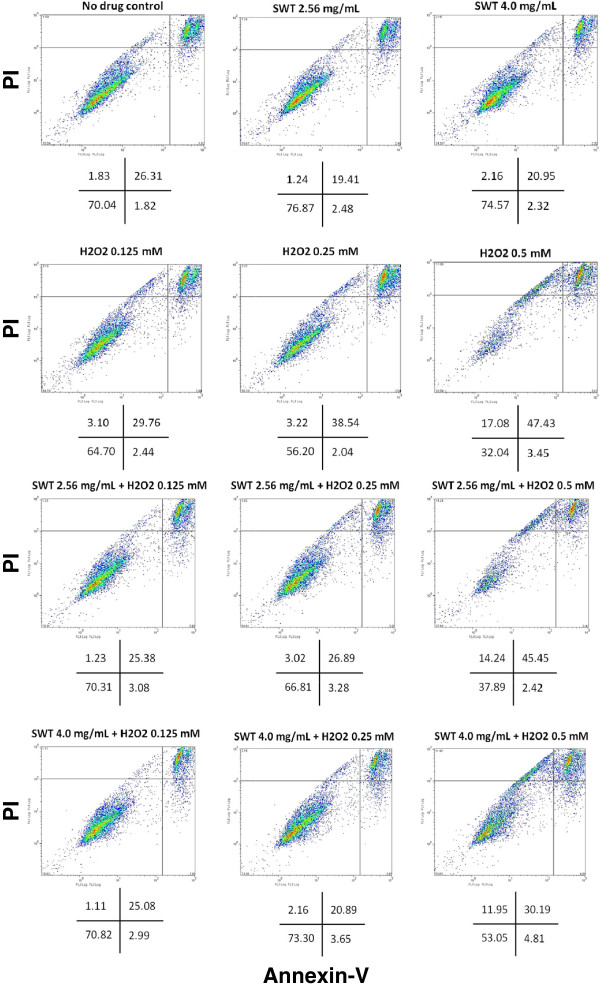
**Effects of SWT on H**_**2**_**O**_**2**_**-induced apoptosis in MCF-10A cells.** MCF-10A cells (3 × 10^5^ cells/well in a 6-well tissue culture plate) were pre-treated with SWT 2.56 or 4.0 mg/mL for two hour then exposed to 0.125, 0.25 or 0.5 mM of H_2_O_2_ for 24 hours. Cells were collected, washed with PBS and resuspended in 1X Annexin V Binding Buffer. Annexin V and/or Propidium iodide (PI) were added to the samples and analyzed by flow cytometry. Flow cytometry profile represents Annexin-V-FITC staining in x axis and PI in y axis. The numbers in each of the quadrant represent the percentage of viable and healthy (lower left), early apoptosis (lower right), necrosis (upper left) and late apoptosis (upper right). The experiment was repeated twice and obtained consistent results.

The protection effect of SWT against H_2_O_2_ – induced apoptosis in MCF-10A cells was examined by the Annexin-V binding and Propidium iodide (PI) staining and flow cytometry analysis. The MCF-10A cells were exposed to various concentrations of H_2_O_2_ (0.125, 0.25, 0.5 mM) alone or in combination with SWT in two concentrations (2.56 and 4.0 mg/mL) for 24 hours. This assay was able to categorize the cell population into four stages: viable and healthy (Annexin and PI-negative), early apoptosis (Annexin-positive/PI-negative), necrosis (Annexin-negative/PI-positive) and late apoptosis (Annexin and PI-positive) (Figure 2). Cells treated with either SWT 2.56 mg/mL or 4.0 mg/mL alone for 24 hours did not show any toxicity, but slightly increased the percentage of viable stage from 70.04% of the control cells to 76.87 and 74.57%, respectively. H_2_O_2_ induced dose-dependent apoptosis. The treatment of 0.125, 0.25 and 0.5 mM H_2_O_2_ resulted in a decrease of viable cells to 64.7%, 56.2% and 32.04%, and an increase in late apoptotic cells to 29.76, 38.54 and 47.43%, respectively. The treatment by SWT results in a dose-dependent increase of viable cells. For example, 4 mg/ml SWT increased the viable cells from 64.70 to 70.82% for the cells treated by 0.125 mM of H_2_O_2_. For the cells treated by 0.25 mM of H_2_O_2_, SWT increased the viable cells from 56.20 to 73.30%. For the cells treated by 0.5 mM of H_2_O_2_, SWT increased the viable cells from 32.04 to 53.05% (Figure 2). Similarly, the treatment by SWT also results in a dose-dependent decrease of cells in the late apoptosis stage (Figure 2).

### Upregulation of Nrf2-dependent genes by SWT

**Figure 3:**
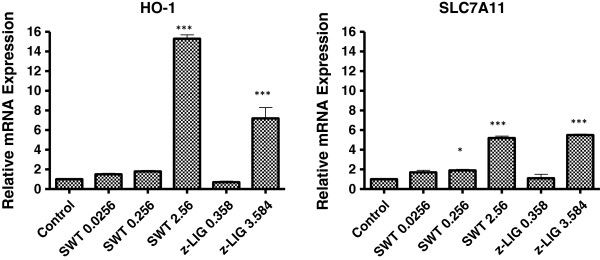
**Upregulation of Nrf2-regulated genes *HMOX-1 *(*HO-1) *and *SLC7A11 *by SWT or z-LIG in MCF-10A cells.** MCF-10A cells were seeded at a density of 2 × 10^5^ cells per well in a 6-well tissue culture plate and treated with SWT or z-LIG for 6 hours. RNA was extracted and quantitative RT-PCR was performed. Data shown are means from three replicates; bars, S.E. *: *p* <0.05; ***: *p* < 0.0001 compared to control.

To determine whether SWT induces any Nrf2-regulated genes, the gene expression of *HMOX-1*, *SLC7A11* and *NQO1* in MCF-10A cells was examined by quantitative RT-PCR. After the cells were treated with SWT in three concentrations (0.0256, 0.256 and 2.56 mg/ml) for 6 hours, the expression of *HMOX-1* and *SLC7A11* was increased in a concentration-dependent manner (Figure 3). The higher dose of SWT (2.56 mg/ml) induced mRNA expression of *HMOX-1* by 15.3 ± 0.4 fold (*p* < 0.01), of SLC7A11 by 5.2 fold ± 0.2 (*p* < 0.0001). The medium dose (0.256 mg/mL) also induced *SLC7A11* by 2.9 ± 0.72 fold (*p* < 0.05). Although SWT also induced expression of *NQO1*, the fold change was not significant (data not shown).

### Upregulation of protein levels of Nrf2 and HMOX-1 by SWT

**Figure 4:**
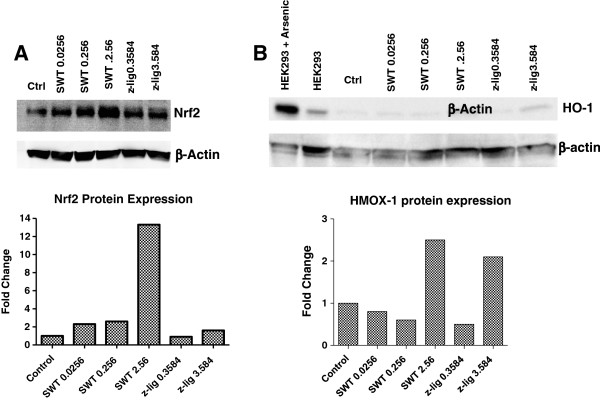
**Upregulation of Nrf2 and HMOX-1 (HO-1) at protein levels by SWT or z-LIG. A.** Western blot analysis of Nrf2 in MCF-10A treated with SWT or z-LIG. **B.** Western blot analysis of HO-1 in MCF-10A treated with SWT or z-LIG. MCF-10A cells were seeded at a density of 5 × 10^5^ cells per well in a 6-well tissue culture plate and treated with SWT or z-LIG for 24 hours. Protein was extracted and ran in polyacrylamide gels. The experiment was repeated at least twice and obtained consistent results. Mean intensity value was normalized to β-actin, then to untreated control. HEK293 cells treated with or without 50 uM Arsenic for 8 hours were used as positive/negative controls (not included in graph).

We next examined the protein level of Nrf2 and Nrf2-dependent protein expression of HMOX-1 in SWT-treated MCF-10A cells after 24 hour treatment. SWT enhanced the levels of Nrf2 protein in a dose-dependent manner, with the highest induction at 2.56 mg/ml (Figure 4A). The HMOX-1 protein levels were also found increased by 2.56 mg/ml SWT (Figure 4B). Thus, the gene expression change of HMOX-1 at protein levels is correlated with those at mRNA levels.

### Increased nuclear translocation of Nrf2 by SWT

**Figure 5:**
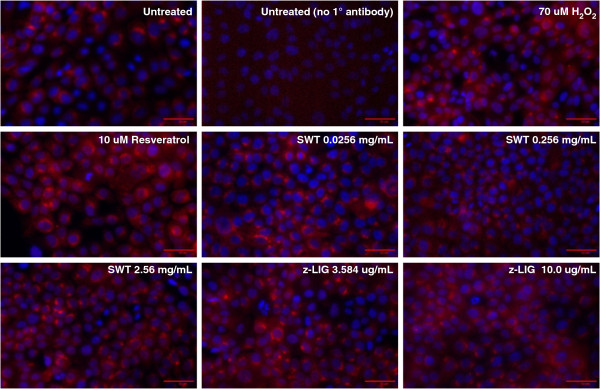
**Increased nuclear translocation of Nrf2 by SWT or z-LIG.** Immunocytochemistry of MCF-10A cells showed dose-dependent nuclear translocation of Nrf2 after treating with SWT or z-LIG for 24 hours. H_2_O_2_ (70 uM) and Resveratrol (10 uM) were used as positive controls. Cells were incubated with Nrf2 antibody (Alexa Fluor 594) after treatment and counterstained with Hoechst (5 uM) for nuclear staining, then visualized under a fluorescent microscope. All images were taken under 20X magnification. Images were merged using ImageJ software (National Institutes of Health). The experiment was repeated twice and obtained consistent results.

To investigate whether SWT can increase the activation of Nrf2 by inducing its nuclear translocation, immunocytochemistry analysis was conducted for MCF-10A cells treated by the SWT. H_2_O_2_ (70 μM) and resveratrol (10 μM) were used as positive controls. The increased nuclear translocation was observed as early as 2 hours treatment (data not shown). SWT dose-dependently induced the translocation of cytoplasmic Nrf2 to the nucleus in MCF-10A after 24 hours treatment, compared to the more diffuse Nrf2 distribution in untreated cells (Figure 5). The translocation of Nrf2 from the cytoplasm into the nucleus by SWT is comparable with that by H_2_O_2_ and resveratrol.

### Induction of Hmox1 and Slc7A11 in liver and mammary glands after oral administration of SWT to rats

**Figure 6:**
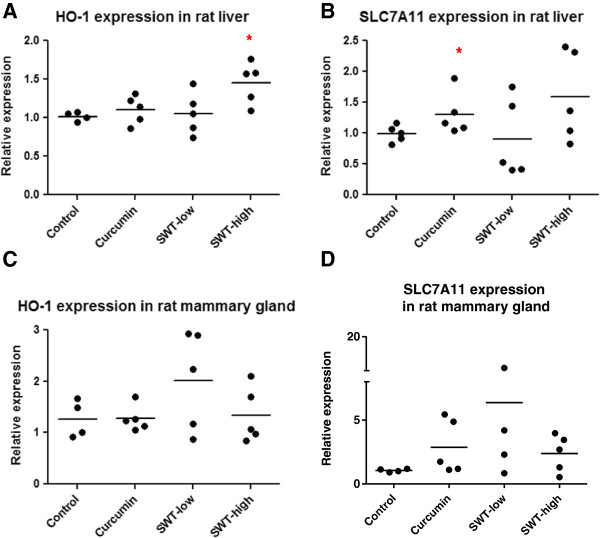
**Induction of *Hmox-1 and Slc7A11 *gene expression in rat liver (A, B) and mammary gland (C, D) after oral administration of curcumin or SWT to the rats.** A daily oral dose of either vehicle control (corn oil), curcumin 200 mg/kg, SWT 250 mg/kg or SWT 1000 mg/kg were administered to female Sprague–Dawley rats (approximately 12 weeks of age, 208 to 215 grams) (n = 5) for six consecutive days. Liver and mammary gland tissues were homogenized and real-time RT-PCR was used to study *Hmox-1* and *Slc7A11* gene expression. *: *p* < 0.05.

The effect of SWT on Nrf2-regulated gene expression was further examined in a short term *in vivo* study on rats. Quantitative RT-PCR assays were used to measure levels of *Hmox-1* and *Slc7A11* transcripts to determine the extent of pharmacodynamic action of SWT in the rat liver and mammary gland. The liver *Hmox-1* gene transcripts were minimally increased in comparison to control following oral administration of curcumin, a positive control (1.1 ± 0.2 fold) and low dose SWT (1.1 ± 0.3 fold) (*p* > 0.05). However, high dose SWT showed significant induction of *HMOX-1* gene (1.5 ± 0.3 fold, *p* < 0.05 compared to control) (Figure 6A). Three out of five rats showed > 1.5-fold increase in *HMOX-1* expression, while none of the rats in the curcumin and low dose SWT groups showed > 1.5-fold increase. The *Slc7A11* gene expression was also induced by high dose SWT or curcumin treatment in rat liver, to 1.6 ± 0.7 fold (*p* = 0.053) and 1.3 ± 0.4 fold (*p* < 0.05), respectively (Figure 6B). The induction of *HMOX-1* and *SLC7A11* by SWT was also observed but not significant in mammary gland possibly due to large variability and small sample size (Figure 6C and D).

### Identification of z-liguistilide (z-LIG) as the active component of SWT

**Figure 7:**
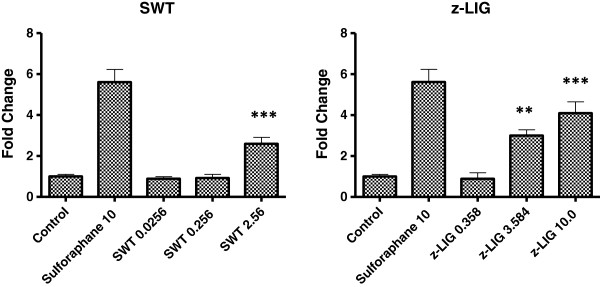
**Induction of ARE luciferase activity by SWT and z-LIG in MCF-7 cells.** The cells were seeded at a density of 1.5 × 10^4^ cells per well in 96-well tissue culture plate and treated with SWT or z-LIG for 24 hours. Data are expressed as fold change, first normalized to the Renilla internal control, then to the untreated control. Data are from three replicates; bars, S.E. **: p < 0.05; ***: *p* < 0.0001 compared to untreated control.

In our previous study, treatment by SWT and its four herbal ingredients increased the ARE-luciferase reporter activity in MCF-7 cells [30]. Consistent results have been seen in MCF-10A cells (data not shown). Probably due to higher transfection efficiency in MCF-7 cells, the results obtained from MCF-7 cells showed higher fold of induction in ARE activity by SWT (data not shown). We decided to use the luciferase reporter gene assay on the MCF-7 cells to identify the active components in SWT that activate the Nrf2 pathway. Based on literature search, nine compounds have been identified in SWT as major components: gallic acid, ligustrazine, paeoniflorin, paeonol, ferulic acid, z- liguistilide, senkyunolide A, butylphthalide, and catalpol [19]. Among the nine compounds tested with a single concentration (10 μg/ml), z-liguistilide (z-LIG) showed the highest activity in the induction of the ARE luciferase activity (data not shown). Therefore, the follow-up experiments were focused on z-LIG. As can be seen in Figure 7, SWT dose-dependently induced the ARE in MCF-7 cells. MCF-7 cells were then treated with various concentrations of z-LIG in triplicates and the luciferase activity was measured (Figure 7). Z-LIG at 3.584 and 10.0 μg/mL increased the ARE activation by 3.0 ± 0.3 and 4.1 ± 0.6 folds, respectively (*p* < 0.05). Because 3.584 μg/mL of z-LIG is equivalent to the amount present in 2.54 mg/mL of SWT, the results obtained from luciferase assay indicates that z-LIG is the main constituent in SWT that contributes to Nrf2 activation. Consistently, z-LIG (3.584 μg/ml) was able to induce mRNA expression of *HMOX-1* and *SLC7A11* in MCF-10A cells (Figure 3). The protein levels of HMOX-1 (Figure 4B) were also increased by z-LIG. Immunocytochemistry data revealed that z-LIG (3.584 and 10 μg/ml) also induced the translocation of cytoplasmic Nrf2 to nucleus in MCF-10A after 24 hours treatment (Figure 5).

## Discussion

One major mechanism for cellular defense against oxidative stress is activation of the Nrf2-antioxidant response element signaling pathway. Nrf2 is the master regulator of a group of genes encoding for proteins that detoxify electrophiles and ROS, and remove or repair the damaged cellular components. Many natural products with chemopreventive activities target Nrf2 pathway by upregulating Nrf2-regulated genes. For example, sulforaphane, allyl isothiocyanate, indole-3-carbinol and parthenolide were found to induce the expression of Nrf2 *in vitro*[31]. Herbal medicines provide a promising source to develop alternative and complimentary medicines modality for cancer prevention. Our previous studies revealed that many detoxifying and antioxidant genes that are under the transcriptional control of the Nrf2 pathway showed significant up-regulation induced by SWT in the breast cancer cell line MCF-7 cells [30]. This suggests that SWT may have the cancer preventive activity through activation of the Nrf2 pathway. However, MCF-7 is a cancer cell line. The chemopreventive effects of SWT need to be examined in non-tumorigenic cell lines. Therefore, we conducted further studies on non-cancerous human breast epithelial cell line MCF-10A for SWT’s effect on H_2_O_2_-induced cytotoxicity and apoptosis and the Nrf2 pathway and confirm our findings in healthy animals. In addition, using the same bioassays for SWT’s activity, we also identified the main active component of SWT that play the role in activating Nrf2.

One strategy in cancer prevention is to protect healthy cells from endogenous or exogenous source of ROS. Oxidative stress can lead to DNA damage, lipid peroxidation, mitochondrial failure and eventually carcinogenesis [32]. The SWT’s antioxidant activity was tested against the effect of H_2_O_2_, a well-known chemical and ROS that induces apoptosis in cells at high concentrations [32]. Some natural products with chemopreventive effect such as genistein and curcumin exhibited a similar protective activity against oxidative stress induced by H_2_O_2_[33,34]. The MCF-10A cells pre-treated with SWT for two hours exhibited cytoprotective effects against H_2_O_2_. The percentage of viable cells increased as demonstrated by an increase of the IC_50_ after SWT treatment compared to H_2_O_2_ treatment alone. A quantitative analysis of apoptosis was performed using the Annexin V and PI staining assay to verify that this protective mechanism is via the anti-apoptotic effect. Our results showed that there were an increase of viable cells and a decrease of apoptotic cells after SWT treatment in combination with H_2_O_2_. This result, for the first time, demonstrates the protective effect of SWT on non-cancerous cells against oxidative stress. However, the protective effect of SWT may result from direct reaction of SWT and H_2_O_2_ in the culture media. We believe that the protective effect of SWT is likely due to a combined mechanisms (directly reaction between SWT and H2O2 and indirectly action via boosting cells’ defense system such as the Nrf2 pathway) because SWT has more than 100 chemical components. Further studies need to be conducted to clarify this issue.

Results from real-time PCR analysis revealed a significant upregulation of selected Nrf2-regulated genes, such as *HMOX-1* and *SLC7A11* after six hours treatment by SWT. Based on Western blot analysis, Nrf2 and HMOX-1 protein expression levels were also upregulated after SWT treatment for 24 hours. Under homeostatic conditions, Nrf2 is sequestered by Keap1 protein in the cytoplasm. Upon activation by chemopreventive agents, Nrf2 dissociates from Keap1 and translocate into the nucleus, which then mediates the induction of phase II detoxifying enzyme genes through binding to ARE [17]. Immunocytochemistry showed that Nrf2 was upregulated and translocated to the nucleus upon SWT treatment. In addition, dual-luciferase assay was used to study the regulatory element ARE after the SWT treatment. Results showed an increase in ARE activation after treating with SWT. Therefore, we conclude that the cytoprotective effect of SWT may be partially mediated through the activation of Nrf2 pathway.

There are nine compounds reported in SWT and five compounds detectable in the batch of SWT product used in our studies [19]. We used the luciferase assay to determine which compounds are the one inducing Nrf2. The concentrations that we selected are based on the corresponding amount of content present in SWT (percent weight) [19]. Z-LIG was identified as the most active compounds inducing Nrf2. However, we cannot rule out the possibility that other components in the SWT may also have the Nrf2 inducing activity. The molecular effects induced by SWT and by z-LIG were not always consistent. For example, the results in Figure 4A showed that HO-1 protein increased to a similar degree after SWT and z-LIG treatment, while Nrf2 protein did not strongly increase after z-LIG treatment. Due to a high level of induction of HO-1 protein by z-LIG (Figure 4B) and induction of the ARE promoter by z-LIG (Figure 7), we still believe z-LIG increased the Nrf2 activity. Although the Nrf2 protein level is regulated by Nrf2 transcription factor, it is also determined by multiple other factors such as ubiquitination and phosphorylation, and thereby its expression may not be consistent with HO-1 and other Nrf2 regulated genes. How z-LIG activates the Nrf2 pathway and whether it shares the same mechanisms of other Nrf2 activators need further investigation. z-LIG may also be a potential chemopreventive agent. Consistently, a published *in vivo* study showed that z-LIG’s anti-oxidant properties increased the level of glutathione peroxidase and superoxide dismutase in ischemic brain tissues and resulted in neuroprotection [35]. In another study, z-LIG was shown to be able to activate Nrf2 and transcription of ARE regulated genes [36].

From our short-term SWT treatment *in vivo* study, we found that *Hmox-1* and *Slc7A11* was upregulated in the liver when the rats were fed 1,000 mg/kg/day SWT for six consecutive days. Curcumin was used as a positive control for its well-known cytoprotective and chemopreventive properties [37,38], but its effect was moderate in our study. Upregulation of *HMOX-1* was also observed in the mammary gland for 250 mg/kg SWT, but due to small number of animals in each group, the difference was not statistically significant. In future *in vivo* chemoprevention studies, long term SWT treatment ranging from six weeks to three to six months is needed [37,39]. Using the same dosages of SWT or the pure compound z-LIG to conduct a long term *in vivo* study may allow us to observe a higher induction of the Nrf2 pathway and chemopreventive efficacy.

## Conclusion

Taken together, our findings strongly suggest that the cytoprotective activity of SWT against hydrogen peroxide-induced oxidative damage might be partially mediated through the activation of Nrf2. Since Nrf2-mediated pathway may prevent cancer or many diseases related to the detrimental effects of ROS, SWT may have a great potential as a safe and orally effective agent for chemoprevention.

## Methods

### Compounds

Hydrogen perioxide (H_2_O_2_) was purchased from Sigma. Z-liguistilide (z-LIG) was purchased from Hong Kong Jockey Club Institute of Chinese Medicine Ltd. (Hong Kong, China). Z-LIG was dissolved in 100% methanol, sonicated at room temperature for 30 minutes and stored in −80°C. Curcumin was purchased from Cayman Chemical Corporation (Catalog # 81025) and mixed in corn oil before oral administration to rats. Sulforaphane was purchased from LKT Laboratories and stock solution was prepared in dimethyl sulfoxide (DMSO) and stored in −20°C.

### Preparation of SWT extracts

SWT was obtained from the School of Pharmacy, Chinese University of Hong Kong. These products were manufactured under GMP condition at the Hong Kong Institute of Biotechnology (Hong Kong, China) according to the protocol described in Chinese Pharmacopoeia 2005 [40] with modifications. SWT solution was prepared fresh from powder right before the experiment in DMEM/F12 complete medium and sonicated at room temperature for 30 minutes. The mixture was centrifuged at 3,000 rpm for four minutes. Cells were treated with the supernatant.

### Cell lines and cell culture

The MCF-7 cells were purchased from American Type Culture Collection (ATCC, Manassas, VA, USA), cultured in Dulbecco’s modified Eagle’s medium (DMEM) supplemented with 10% fetal bovine serum (FBS), 1% non-essential amino acids, 100 unit/mL penicillin, 100 μg/mL streptomycin, 1 mM sodium pyruvate, and 2 mM L-glutamine in an atmosphere of 5% CO_2_ at 37°C. The human breast MCF-10A cell line was obtained from ATCC and maintained in Dulbecco’s modified Eagle’s medium/F12 containing 5% horse serum, EGF 20 ng/mL, hydrocortisone (0.5 mg/mL), cholera toxin (100 ng/mL), insulin (10 ug/mL) and 1% penicillin/streptomycin.

### Sample preparation for RNA extraction

MCF-10A cells seeded in 6-well plates at a density of 2.5 × 10^5^ cells per well were treated with SWT or z-LIG for six hours. RNA was extracted using RNeasy Plus Mini Kit (Qiagen) following manufacturer’s protocol. Rat mammary gland and liver tissues were homogenized in TRIzol Reagent (Invitrogen) using a large handheld electric homogenizer (Polytron, Kinematica Ag) to extract total RNA. Mammary gland tissues along with part of skin and fur were homogenized and used for RT-PCR analysis.

### Real-time RT-PCR

cDNA was synthesized using the High Capacity cDNA Reverse Transcription Kit (Applied Biosystems). Real-time PCR was carried out using the Power SYBR Green PCR master mix (Applied Biosystems) or the RT^2^ Real-Time SYBR Green/Rox PCR master mix (SA Biosciences). Both master mixes were validated to yield similar results. PCR programming was as followed: 50°C for 2 minutes; 95°C for 10 minutes; 40 cycles of 95°C for 15 seconds, 60°C for 1 minute; 95°C for 15 seconds, 60°C for 1 minute, 95°C for 15 seconds and 60°C for 15 seconds. The primer sequences are available upon request.

### Cell proliferation assay

Sulforhodamine B (SRB), a protein-binding reagent (Sigma), was used in the cell proliferation assay to determine drug potency. In each experiment, 3,000 to 4,000 cells per well were seeded in 96-well plates and allowed to attach overnight. Cells were treated with SWT or compounds in triplicates or six replicates for 24 hours and incubated at 37°C in 5% CO_2_/95% air. Cells were pre-treated with SWT for two hours before addition of H_2_O_2_ in the case of combination treatments. Cell viability and IC_50_ were determined by averaging the triplicate or six replicates of the readings and normalizing to untreated control. Dose–response curves were plotted using Prism software (San Diego, California).

### Luciferase reporter gene assay

The luciferase reporter construct pGL4.22-ARE was a gift from Dr. Donna Zhang at University of Arizona. It was generated by cloning a 39-bp ARE-containing sequence from the promoter region of the NAD(P)H quinone oxidoreductase 1 (NQO1) into the pGL4.22 vector (Promega, Madison, WI) [41]. The MCF-10A or MCF-7 cells were transfected with the pGL4.22-ARE plasmid and a constitutively active renilla luciferase (pRL-TK-luc, from Promega; to correct for tranfection efficiency) (40:1 ratio) using FuGENE HD Transfection Reagent (Roche Applied Science, Indianapolis, IN) according to the manufacturer’s instructions. Twenty-four hours after transfection, the cells were exposed to the extracts of SWT or components for another 24 hours. Cell lysates were used for determining luciferase activities of both firefly and renilla by the dual luciferase reporter gene assay (Promega). Firefly luciferase activity was normalized to renilla luciferase activity. The experiment was carried out in triplicate and expressed as the mean ± SD.

### Western blot analysis

MCF-10A cells were seeded in 6-well tissue culture plates at a density of 5 × 10^5^ cells per well. Cells were then treated with SWT or z-LIG for 24 hours. 293 (HEK) cells treated with 50 μM Arsenic for 8 hours (lysate provided by Cell Signaling) was used as a positive control. The protein concentration was determined using the BCA protein assay kit (Pierce Chemical Company) according to manufacturer’s protocol. Primary antibodies were prepared in either 5% BSA or milk and blots were incubated for 18 hours in 4°C. The following concentrations for primary antibodies were used: β-actin 1:500 (Millipore), Nrf2 1:1000 (Cell Signaling) and HMOX-1 1:200 (Cell Signaling). Blot was incubated with secondary antibody for 90 minutes at a 1:2000 or 1:3000 dilution prepared in 5% milk. Western blot Luminol Reagent (Santa Cruz) was used for chemiluminescence detection.

### Immunocytochemistry

MCF-10A cells were seeded at a density of 5 × 10^4^ cells per well in 24-well tissue culture plates and treated with SWT or z-LIG for 24 hours. Resveratrol 10 uM and H_2_O_2_ 70 uM were used as a positive controls. After treatment, cells were washed twice with warm 1X PBS before fixing with 3.7% ultrapure formaldehyde solution (at 37°C) and incubated for 15 minutes at room temperature. Cells were then washed four times with 1X PBS and permeabilized with 0.2% triton X-100, washed and blocked with 2.5% BSA for 60 minutes. Cells were then incubated overnight in 4°C with primary antibody Nrf2 (Cell Signaling) at a dilution of 1:200 prepared with 2.5% BSA in 1X PBS. Cells were washed four times with PBS ± 0.05% Tween-20 before addition of secondary antibody (Alexa-fluor dye) at 1:1000 dilution for 60 minutes at room temperature in the dark. Cells were counterstained with Hoechst 5 μM to show nuclear morphology.

### Animal studies

Female Sprague–Dawley rats (approximately 12 weeks of age, 208 to 215 grams) were purchased from Harlan Laboratories. Twelve-week-old rats have relatively mature mammary glands and contain the highest known levels of more differentiated alveolar buds [42]. They were kept in an environmentally controlled breeding room (12 hour dark/light cycle) for one week before experiments began. They were fed standard laboratory chow with water ad libitum. Animals were randomly divided into four groups of five animals each. Each rat received an oral dose of drug or corn oil each day for six consecutive days. Control group received the vehicle alone (0.5 mL of corn oil). Curcumin, used as positive control, was administered at 250 mg/kg/day. SWT low and high dose was 250 and 1,000 mg/kg/day, respectively. These doses were determined from previous studies for SWT on other disease models [22,25,26]. Animals were sacrificed approximately 16 hours after the last dose. Blood samples from each rat were collected through cardiac puncture. Mammary gland tissues were removed, washed with PBS and weighted, then immediately placed in −80°C for further analysis. Tissues used for real-time RT-PCR assay were soaked in RNAlater solution overnight in 4°C before removing the solution and freezing the samples in −80°C. All animal studies were according to the recommendation of the Regulations for the Administration of Affairs Concerning Experimental Animals.

## Abbreviations

SWT: Si-Wu-Tang; HO-1: Heme oxygenase 1; z-LIG: z-liguistilide; ER: Estrogen receptor; ROS: Reactive oxygen species; Nrf2: Nuclear factor erythroid 2 -related factor 2 (Nrf2); ARE: Antioxidant response elements; Keap1: Kelch-like ECH-associated protein 1; PI: Propidium iodide; SRB: Sulforhodamine B; NQO1: NAD(P)H quinone oxidoreductase 1.

## Competing interests

The authors declare that they have no competing interests.

## Authors’ contributions

ML carried out most of the experiments and drafted the manuscript; RR carried out the in vivo studies; ZW assisted the in vitro and vivo studies; ZZ participated in the design of the studies and provided the herbal extracts; MC participated in the design of the studies; AT assisted in the protein level studies; SP participated in the design of the studies; BA performed the statistical analysis; YH conceived the studies, coordinated the experiments and was involved in the drafting the manuscript and revising it. All authors read and approved the final manuscript.
